# Immunotherapy-induced neutralizing antibodies disrupt allergen binding and sustain allergen tolerance in peanut allergy

**DOI:** 10.1172/JCI164501

**Published:** 2023-01-17

**Authors:** Nicole A. LaHood, Jungki Min, Tarun Keswani, Crystal M. Richardson, Kwasi Amoako, Jingjia Zhou, Orlee Marini-Rapoport, Hervé Bernard, Stéphane Hazebrouck, Wayne G. Shreffler, J. Christopher Love, Anna Pomes, Lars C. Pedersen, Geoffrey A. Mueller, Sarita U. Patil

**Affiliations:** 1 Food Allergy Center and Center for Immunology and Inflammatory Diseases, Massachusetts General Hospital, Boston, Massachusetts, USA.; 2 National Institute of Environmental Health Sciences, Research Triangle Park, North Carolina, USA.; 3 InBio, Charlottesville, Virginia, USA.; 4 Harvard University, Cambridge, Massachusetts, USA.; 5 Université Paris Saclay, CEA, INRAE, Département Médicaments et Technologies pour la Santé (DMTS), Gif-sur-Yvette, France.; 6 Koch Institute for Integrative Cancer Research, Massachusetts Institute of Technology, Broad Institute of MIT and Harvard, Cambridge, Massachusetts, USA.

**Keywords:** Immunology, Allergy, Immunoglobulins, Tolerance

## Abstract

In IgE-mediated food allergies, exposure to the allergen activates systemic allergic responses. Oral immunotherapy (OIT) treats food allergies through incremental increases in oral allergen exposure. However, OIT only induces sustained clinical tolerance and decreased basophil sensitivity in a subset of individuals despite increases in circulating allergen-specific IgG in all treated individuals. Therefore, we examined the allergen-specific antibodies from 2 OIT cohorts of patients with sustained and transient responses. Here, we compared antibodies from individuals with sustained or transient responses and discovered specific tolerance-associated conformational epitopes of the immunodominant allergen Ara h 2 recognized by neutralizing antibodies. First, we identified what we believe to be previously unknown conformational, intrahelical epitopes using x-ray crystallography with recombinant antibodies. We then identified epitopes only recognized in sustained tolerance. Finally, antibodies recognizing tolerance-associated epitopes effectively neutralized allergen to suppress IgE-mediated effector cell activation. Our results demonstrate the molecular basis of antibody-mediated protection in IgE-mediated food allergy, by defining how these antibodies disrupt IgE-allergen interactions to prevent allergic reactions. Our approach to studying the structural and functional basis for neutralizing antibodies demonstrates the clinical relevance of specific antibody clones in antibody-mediated tolerance. We anticipate that our findings will form the foundation for treatments of peanut allergy using neutralizing antibodies and hypoallergens.

## Graphical abstract

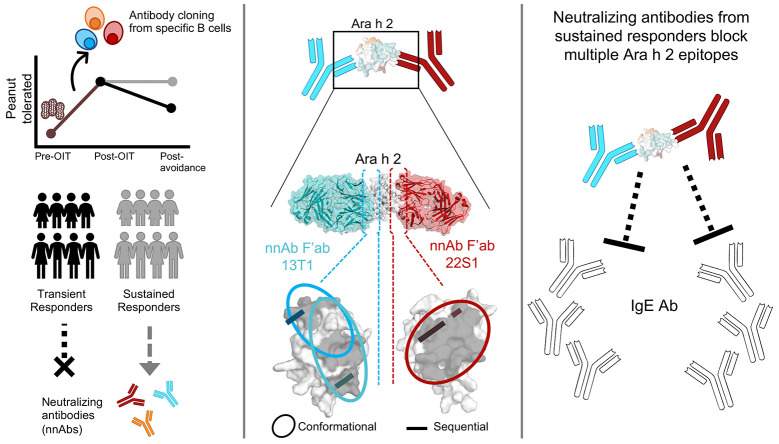

## Introduction

Food allergy affects up to 10% of the US population (1, 2), and more than 40% of those affected have experienced life-threatening anaphylaxis (1). Peanut is one of the most common food allergens, often leading to persistent IgE-mediated food allergy (3, 4). In the past few years, the scientific community has made tremendous strides in preventing and treating IgE-mediated food allergies, including peanut allergy. For instance, the development of peanut oral immunotherapy (OIT), involving clinically monitored increases in oral exposure to peanut, have been developed and approved for treatment of IgE-mediated peanut allergy (5). Despite these advances, understanding long-term sustained tolerance in allergic individuals has been elusive.

Ingestion of allergens either early in life as a preventive measure (6) or through OIT as treatment for food allergies increases circulating allergen-specific IgG and IgG4 levels (7, 8). This induced IgG blocks IgE-mediated activation of effector cells in both peanut-sensitized and -tolerant children (9–11) as well as in OIT-treated peanut-allergic individuals while on therapy (12–14). Only a subset of individuals with peanut allergy, however, develop sustained responses to OIT after OIT is stopped (8, 13, 15). Furthermore, allergen-specific IgG and IgG4 levels increase in all treated individuals, regardless of the clinical efficacy of the treatment (8, 13). Despite this response, only patients with sustained responses after OIT exhibit decreased sensitivity in allergen-induced activation of basophils, which are circulating allergic effector cells (13). Additionally, the decrease in basophil sensitivity begins early in OIT, occurs simultaneously with an increase in circulating allergen-specific IgG, IgG4, and IgA antibodies, and continues after OIT in patients with sustained clinical responses to OIT but not in those with transient responses. This induction of functionally suppressive IgG antibodies is also demonstrated in other forms of immunotherapy (12, 16, 17). Since functional suppression by allergen-specific antibodies, but not levels of allergen-specific antibodies, correlates with clinical efficacy of OIT, we hypothesized that the allergen-specific antibody repertoires induced during OIT may underlie clinical efficacy. However, the molecular determinants of allergen-antibody interactions and their relationship to functional IgE suppression remain unclear.

We aimed to elucidate the mechanism of allergen-specific antibody-mediated tolerance in IgE-mediated food allergy. We used patient samples from 2 clinical trials of peanut OIT designed to determine whether the patients had sustained or transient responses to treatment, by comparing tolerance to peanut directly after OIT and then after 1–3 months of avoidance of OIT. We focused on Ara h 2 because it is the immunodominant, most clinically relevant peanut allergen (18–20). Ara h 2 is a 2S albumin seed storage protein that is an 18 kDa protein composed of 4 α-helices (21). This small protein is remarkably resistant to proteolytic and enzymatic digestion, resulting in the likely preservation of conformational epitopes (22). Using a highly specific fluorescent multimer to Ara h 2, we selected allergen-specific B cells (7) and cloned allergen-specific antibodies to dissect the epitope-paratope structural interactions related to functional allergen neutralization and clinical tolerance.

## Results

### Sustained responses after OIT associate with functional IgE blocking, and not allergen-specific IgG levels.

The 2 clinical trials of peanut OIT here included both children and adults (7, 23), and we stratified outcomes on the basis of the durability of their clinical response. We categorized the participants as sustained or transient responders on the basis of whether they retained tolerance to the same amount or a lower amount of peanut after a period of avoidance. In both combined clinical trials, we identified 19 sustained and 30 transient responders.

To evaluate serum antibody responses, for the first time, we combined longitudinal data from both clinical trials, focusing on the durability of the clinical response achieved after OIT. Similar to other reports, we confirmed that Ara h 2–specific IgG and IgG4 levels increased significantly during peanut OIT, from before (baseline [BL]) to post OIT (POIT), and remained persistently elevated in both sustained and transient responders post avoidance (PAV) (Supplemental Figure 1, A and B; supplemental material available online with this article; https://doi.org/10.1172/JCI164501DS1). Serum levels of Ara h 2–specific IgE were significantly higher in transient responders throughout OIT (Supplemental Figure 1C). Using direct ex vivo basophil activation of peripheral blood from OIT-treated individuals, we found a significant decrease in basophil sensitivity to Ara h 2 in sustained, but not transient, responders (Supplemental Figure 1D; measured by the ED_50_ of the Ara h 2 dose-response curve). Taken together with previously published data, we hypothesized that the induction of specific Ara h 2 antibody clones with enhanced protective functionality may have induced the significant decrease in Ara h 2–specific basophil sensitivity in the sustained responders, despite similar levels of Ara h 2–specific IgG and IgG4 in all participants.

### Identification of Ara h 2 epitope bins.

To test this hypothesis, we sought to compare the epitope and paratope repertoires in sustained and transient responders. We used a fluorescent multimer for isolation of Ara h 2–specific B cells from peripheral blood. We chose 2 time points with the highest frequency of allergen-specific B cells based on previous studies (7), 1–3 months after the start of OIT and 3 months after the PAV food challenge. We amplified 1,671 heavy chains and 395 light chains with 389 paired heavy and light chains from individual Ara h 2–specific B cells (Supplemental Figure 2B). We cloned 74 Ara h 2–specific antibodies from a subset of OIT-treated individuals, 10 sustained responders, and 8 transient responders (Supplemental Table 1).

A subset of these antibodies (*n* = 39) was evaluated by an Ara h 2 peptide microarray (Supplemental Figure 3). Of these antibodies, 16 bound to sequential, or linear, epitopes. We identified 4 main sequential epitopes within Ara h 2. The most abundantly recognized sequence motif, ^63^DPYSP^OH^S^68^, is located on the loop structure between helixes 2 and 3 of Ara h 2 and contains a hydroxyproline. The 3 other sequential epitopes determined were ^30^DRRCQSQLER^39^, ^123^QGRQQEQQ^129^, ^130^KRELRNLPQQ^139^, and in order of decreasing frequency of recognition. We subsequently used biotinylated peptides in biolayer interferometry (BLI) assays to further identify 9 additional antibodies binding to these sequences (Supplemental Table 2). In total, we identified 23 antibodies specific to sequential epitopes.

**Figure 1:**
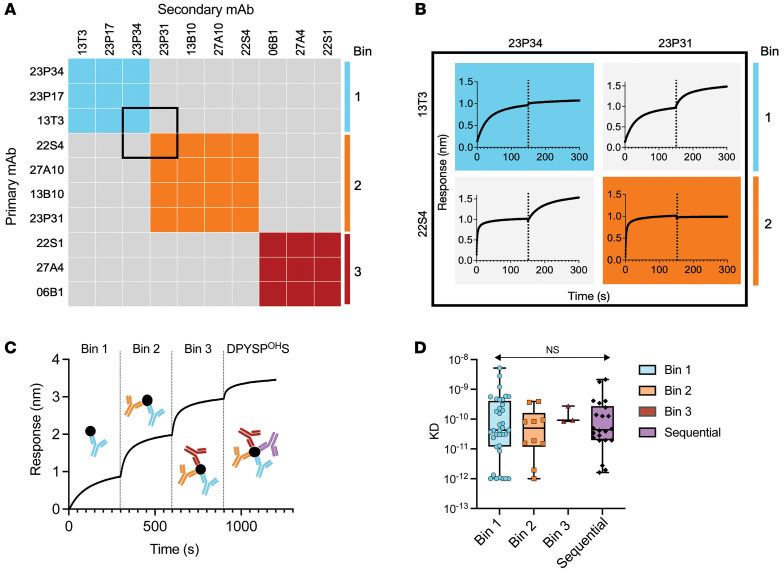
Conformational epitope binning of Ara h 2–specific antibodies. (**A**) Representative epitope binning of nonsequential epitope Ara h 2 antibodies with an in-tandem epitope binning assay on BLI identified 3 conformational bins (colored boxes). The average response for binding of secondary binding antibodies was 0.77 ± 0.25 nm compared with 0.04 ± 0.05 nm for nonbinding secondary antibodies. (**B**) Inset with raw biolayer sensograms of the pairwise comparisons of primary (left) and secondary (top) Ara h 2–specific mAb binding to immobilized native Ara h 2. Primary and secondary antibody associations are divided by black dotted lines. (**C**) Sequential binding of epitope-specific antibodies to immobilized native Ara h 2. (**D**) Affinity of Ara h 2 antibodies for native Ara h 2 measured by BLI (shading by epitope). KD, dissociation constant. Box-and-whisker plots represent the mean, quartiles, and range. Statistical comparison was performed by 2-way ANOVA.

We evaluated the potential number of conformational epitopes of the remaining 49 antibodies that did not bind to linear peptides by microarray or BLI. For epitope binning, we used in-tandem antibody assays with immobilized native Ara h 2 on BLI. Secondary antibodies were grouped into a separate epitope bin if binding was 0.16 nm or greater (≥4 times the SD of nonbinding antibodies). We identified 3 conformational epitope bins for Ara h 2 that we designated as bins 1, 2, and 3 (Figure 1, A and B).

We next sought to determine whether these bins had significantly overlapping epitopes and whether they were separate from the unique Ara h 2 loop, which contains the sequential epitope DPYSP^OH^S. Using a sequential binding assay to immobilized native Ara h 2 on BLI, we determined that the 3 conformational epitopes and the most common sequential epitope, DPYSP^OH^S, bind to distinct, nonoverlapping areas of Ara h 2 (Figure 1C).

### All Ara h 2 mAbs have similar high affinity.

We then measured the affinity of the mAb for Ara h 2 by BLI (Supplemental Figure 4). Overall, 69 of the 74 antibodies (93%) had a subnanomolar affinity (<1 × 10^-9^ nM) for Ara h 2. There was no significant difference between the affinity of antibodies that recognized the 3 different epitope bins or sequential epitopes. (Figure 1D).

### Sustained responders have mAbs targeting all 3 bins.

**Figure 2:**
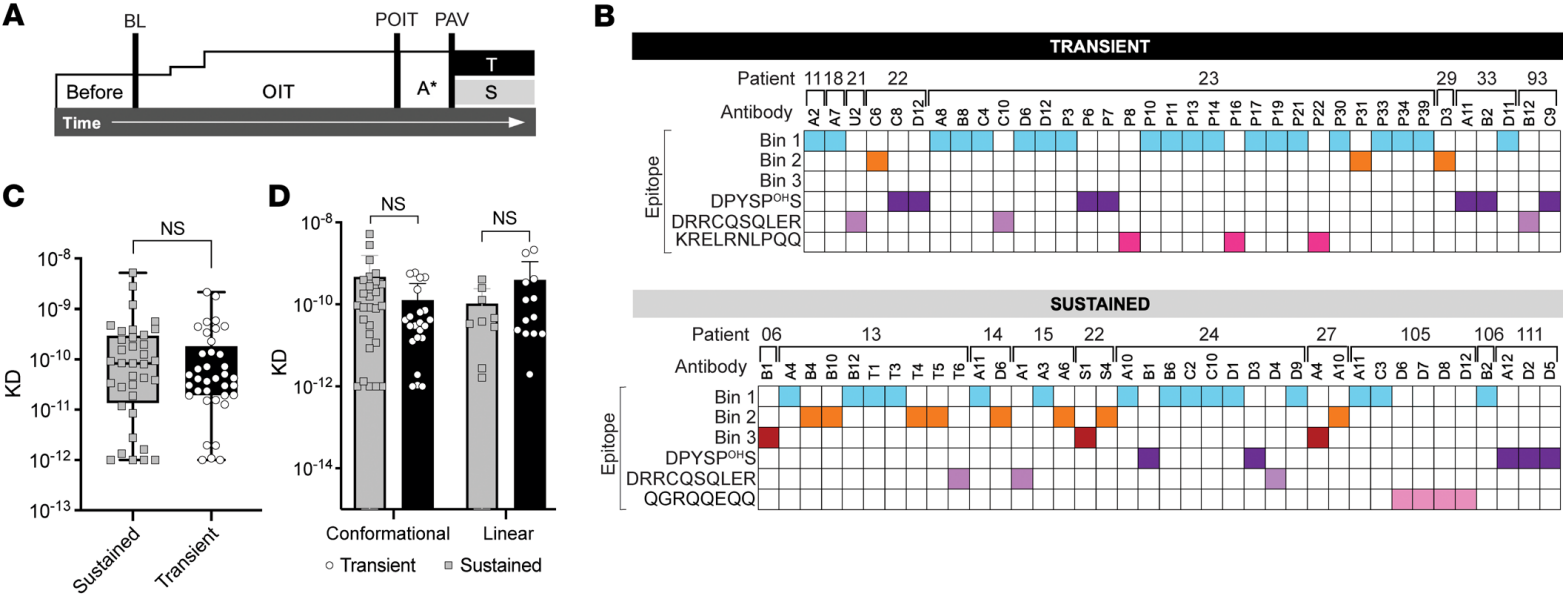
Characterization of high-affinity Ara h 2 epitope–specific antibodies from OIT-treated individuals. (**A**) Clinical schema of peanut OIT, where tolerance to peanut after 1 year of OIT (POIT) followed by tolerance PAV (A* 1–3 months) led to classification as sustained (S) or transient (T) responders. (**B**) Epitope specificity of mAbs from peanut OIT. (**C** and **D**) Affinity of Ara h 2 antibodies for native Ara h 2 measured by BLI (sustained responders are shown in gray and transient responders in black). Box-and-whisker plots represent the mean, quartiles, and range. Statistical comparison was performed with a Mann-Whitney *U* test.

We next sought to evaluate whether we could identify differences in bin recognition between Ara h 2 antibodies from different clinical outcomes of OIT. From the 2 clinical trials of OIT, we performed recombinant cloning of antibodies from sustained (*n* = 38 antibodies) and transient responders (*n* = 36 antibodies) (Figure 2A). Using both sequential epitope mapping and conformational epitope binning, we identified mAbs recognizing both sequential and conformational bins in both sustained and transient responders (Figure 2B).

Overall, 50% of the antibodies (*n* = 20 from transient and *n* = 17 from sustained responders) bound to bin 1, which appeared to be immunodominant. The three bin 3 antibodies were the rarest antibodies and were only identified in sustained responders.

We also compared the affinity of antibodies from sustained and transient responders. There was no significant difference in the affinity for Ara h 2 of mAbs from sustained or transient responders (Figure 2C). Also, there was no significant difference in affinity when comparing sequential and conformational epitope–binding antibodies (Figure 2D).

We next hypothesized that structural determinants of antigen-antibody interactions significantly influenced the protective functionality of Ara h 2–specific antibodies.

### Localization and crystallization of conformational Ara h 2 epitopes.

**Figure 3:**
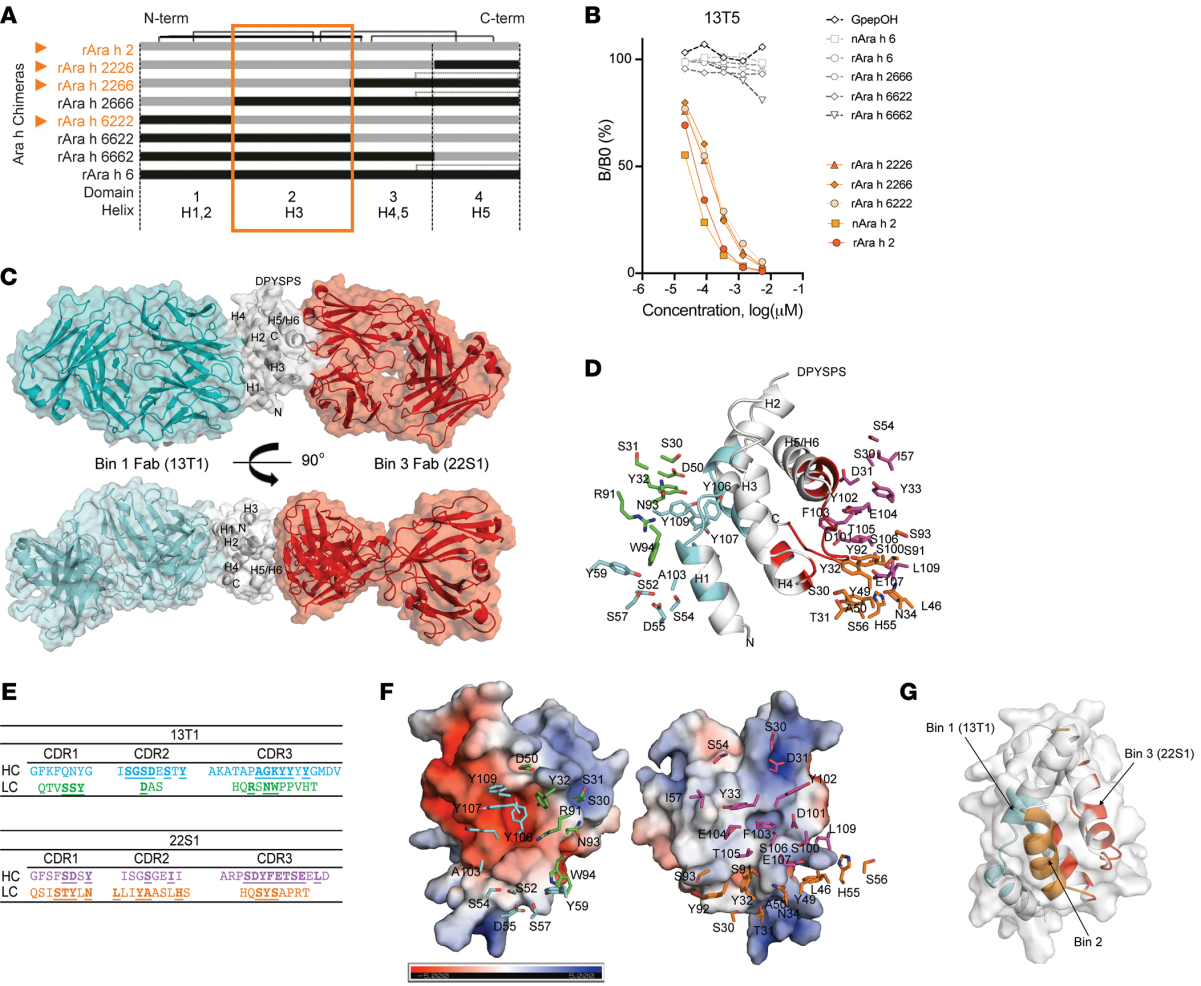
Definition of Ara h 2 conformational epitopes. (**A**) Schema of Ara h 2 and Ara h 6 chimeras used in a (**B**) competitive inhibition ELISA assay to evaluate binding to the mAb 13T5 (orange represents the Ara h 2 domain 2 containing chimeras). The chimera name denotes Ara h 2 or Ara h 6 α-helices that the chimera contains in domain positions 1–4. (**C**) Overall arrangement of the ternary complex with bin 1 Fab (13T1, cyan), bin 2 Fab (22S1, red), and Ara h 2 (white) by x-ray crystallography. (**D** and **E**) Paratope residues interacting with Ara h 2 at the interface. The heavy and light chain carbons of 13T1 are shown in cyan and green; 22S1 heavy and light chain carbons are depicted in magenta and orange, respectively. Interacting residues are underlined. (**F**) Electrostatic surface properties of Ara h2 demonstrating Y106 (13T1) and F103 (22S1) packing against negative and positive pockets, respectively (related to Supplemental Table 3). Heavy and light chain carbons are colored as in **E**. (**G**) Ribbon representation of the 3 conformational epitopes of Ara h 2 (cyan shading for 13T1 bin 1, orange shading for bin 2, and red shading for 22S1 bin 3), with gray surface representation.

Once we identified 3 distinct, nonoverlapping epitope bins, we sought to localize them using chimeric proteins with key sequential differences that might modify antibody recognition. We used another peanut 2S albumin, Ara h 6, which shares 59% homology with Ara h 2 (21, 24). Chimeric proteins were designed by splitting the sequences into 4 domains, each containing 1 or 2 of the α-helices of these proteins. These Ara h 2 and Ara h 6 elements were then spliced together to create well-folded chimeric proteins exhibiting the conformational characteristic of the 2S-albumins (Figure 3A). We then used a competitive inhibition ELISA to test mAb binding to these chimeric constructs. Bin 2 conformational antibodies bound only to those chimeras with the Ara h 2 domain 2, corresponding to Ara h 2 α-helix 3 (Figure 3B). Bin 3 antibodies bound to all chimeras, and bin 1 antibodies bound inconsistently to domains 1 and 3 (Supplemental Figure 5, A–C). In summary, the chimeras localized bin 2 antibody recognition to helix 3 of Ara h 2.

To refine the locations of bins 1 and 3, we used x-ray crystallography. The ternary complex of Ara h 2 bound to Fab fragments from antibodies in conformational bins 1 and 3 (13T1 and 22S1, respectively) was solved (Supplemental Table 3), revealing the 2 nonoverlapping bins residing on opposite sides of the Ara h 2 structure (Figure 3C). In the 13T1Fab–Ara h 2–22S1Fab ternary complex, these 2 bins were nonoverlapping and bound at sites located almost 180 degrees from one another on Ara h 2.

The bin 1 epitope space covers the N-terminus of Ara h 2 containing helixes 1, 2, and 4, while bin 3 covers the C-terminus surrounded by helixes 4 and 5, respectively. Bin 1 and 3 epitope residues form multiple hydrogen-bonding interactions and salt bridges with residues on complementarity-determining regions (CDRs) of 13T1 and 22S1 (Figure 3D).

Among them, the most contacts were made by residues on the CDR 3 of the heavy chain (CDRH3) (Figure 3E). One noticeable feature at the interface is the tight packing of key residues against a charged groove formed by the helices and loop of Ara h 2. The side chain of the Y106 of the 13T1 CDRH3 packs against the negatively charged groove formed by helixes 1, 2, and 4, whereas the F103 of the 22S1 CDRH3 snugly fits into the positively charged pocket formed by helix 5 and the C-terminus (Figure 3F). Combining the results from x-ray crystallography and chimeric domain 2 data, we localized all 3 nonoverlapping conformational bins of Ara h 2 (Figure 3G).

### Ara h 2 antibody epitopes specific to sustained responders after OIT.

**Figure 4:**
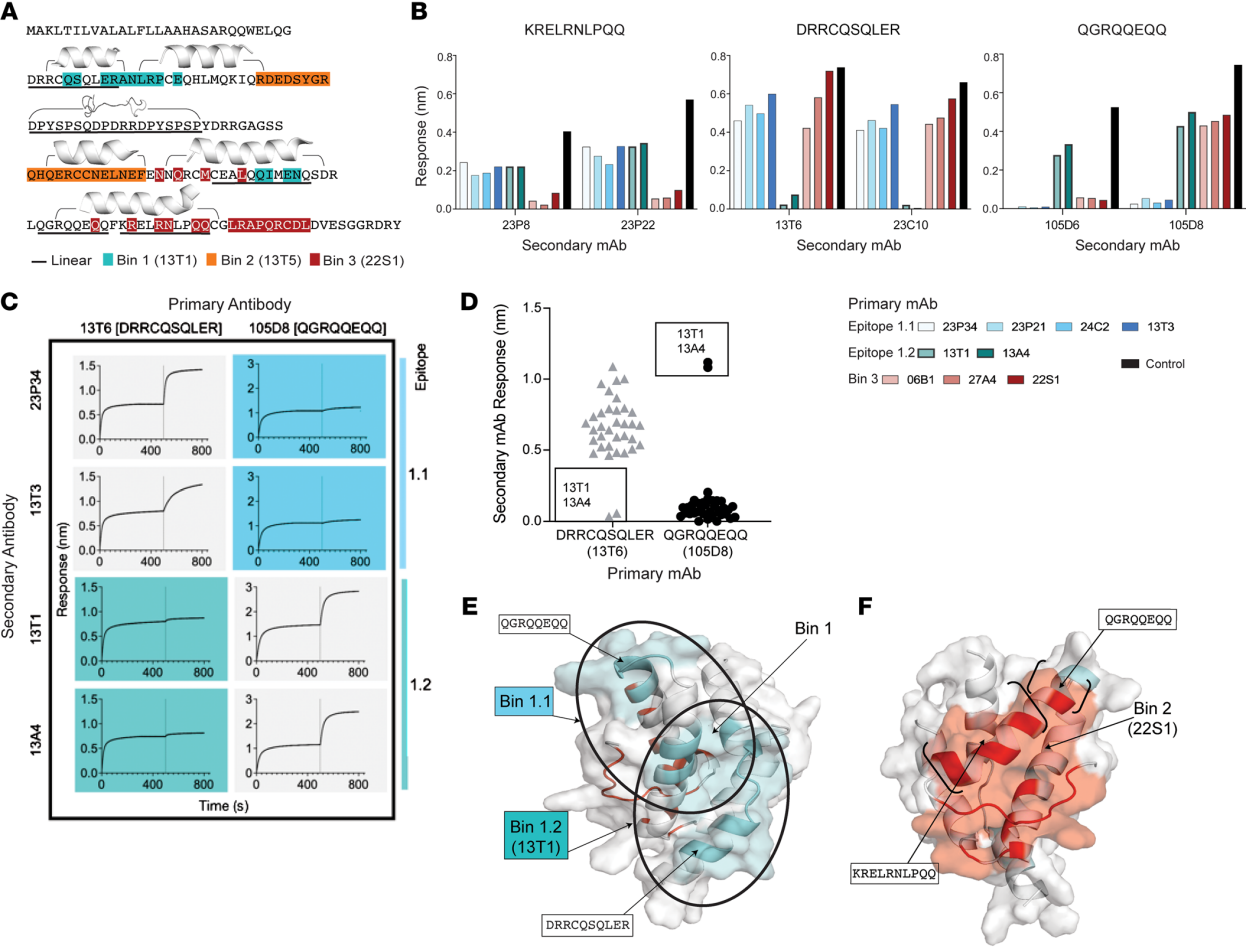
Identification of bin 1 and 3 epitopes specific to sustained responders in OIT. (**A**) Protein sequence of Ara h 2.0201 with shading conformational residues identified by x-ray crystallography on Ara h 2.0101 (13T1 in teal, 22S1 in red), chimeric proteins (bin 2 in orange), sequential epitopes (underlined), and helices (bracketed). (**B**) Epitope binning with primary bin 1 or 3 conformational antibodies and secondary antibodies recognizing sequential epitopes. (**C**) Biolayer sensorgrams using primary antibodies (top) recognizing sequential epitopes before binding of secondary bin 1 conformational mAbs (left) identified two bin 1 overlapping epitopes (colored boxes). (**D**) Response rates of secondary conformational bin 1 antibodies after binding of primary DRRCQSQLER and QGRQQEQQ antibodies. (**E** and **F**) Ribbon representations of overlapping epitopes 1.1 and 1.2 (blue) and bin 3 (red) with sequential epitopes (bracketed) overlaid with surface representation.

Using the structural epitope maps of bin 1 and bin 3, we found sequential epitopes overlapping with conformational bins 1 and 3. The 13T1 epitope (bin 1) contains 4 overlapping amino acids with the sequential epitope ^30^DRRCQSQLER^39^ (Figure 4A). The 22S1 epitope (bin 3) contains the 5 overlapping amino acids with epitope ^130^KRELRNLPQQ^139^ and 1 overlapping amino acid with epitope ^121^QGRQQEQQ^128^ (Figure 4A). We hypothesized, given the localization of these epitopes, that antibodies recognizing conformational epitopes that also overlap with sequential epitopes may more effectively disrupt IgE binding to Ara h 2.

First, in a BLI assay, we tested whether bin 1 and bin 3 mAbs could block the binding of antibodies that recognized their respective overlapping sequential epitopes. As expected, we found that antibodies recognizing ^130^KRELRNLPQQ^139^ and ^121^QGRQQEQQ^128^ were blocked by bin 3 antibody binding to Ara h 2 (Figure 4B). On the other hand, some bin 1 antibodies blocked ^30^DRRCQSQLER^39^ and some blocked ^121^QGRQQEQQ^128^ (Figure 4B), suggesting that there may be 2 epitopes within bin 1.

To localize these epitopes within bin 1, we then tested the ability of bin 1 antibodies to bind to Ara h 2 after antibodies recognizing epitopes ^30^DRRCQSQLER^39^ and ^121^QGRQQEQQ^128^ were bound to Ara h 2 in a BLI assay (Figure 4C). The difference in response rates of the bin 1 antibodies after blocking of these sequential epitopes by mAbs identified 2 overlapping bin 1 epitopes of Ara h 2 (Figure 4D). We named these overlapping epitopes 1.1 and 1.2. Antibodies against epitope 1.1 are blocked by antibodies against the ^121^QGRQQEQQ^128^ epitope, and antibodies against epitope 1.2 are blocked by antibodies against the ^30^DRRCQSQLER^39^ epitope (Figure 4E). Interestingly, epitope 1.2 only contains antibodies from sustained responders, including 13T1.

In support of these findings, these 2 epitopes in bin 1 also have different patterns of binding to the chimeric proteins. The epitope 1.1 antibody 13T1 binds to chimeras containing domain 1 of Ara h 2 in distinct comparison with epitope 1.2 antibodies, which all bind chimeras containing domain 3 of Ara h 2 (Supplemental Figure 5A).

Antibodies recognizing epitope 1.2 have several unique characteristics. These antibodies have uniquely long heavy and light chain CDR3s compared with the more commonly identified 1.1 epitope (Supplemental Figure 6, A and B). Second, the 13T1 antibody that binds within these sustained response-associated epitopes is highly similar to previously identified IgE antibodies from a separate cohort of individuals with peanut allergy (Supplemental Figure 6B). The Y106 in 13T1 appears highly conserved in the CDRH3 alignment with these public antibodies, likely due to clonal convergence.

Here, we identified epitopes 1.2 and 3, which were exclusively recognized by antibodies from sustained responders (Figure 4, E and F). Overall, our structural studies of these epitopes demonstrated their unique localization and ability to disrupt allergen binding by antibodies recognizing sequential epitopes. We hypothesized that the unique epitope recognition patterns of antibodies from sustained responses confer their capacity to effectively block IgE cross-linking and possibly clinical protection.

### Neutralizing Ara h 2–specific antibodies inhibit IgE binding to Ara h 2.

**Figure 5:**
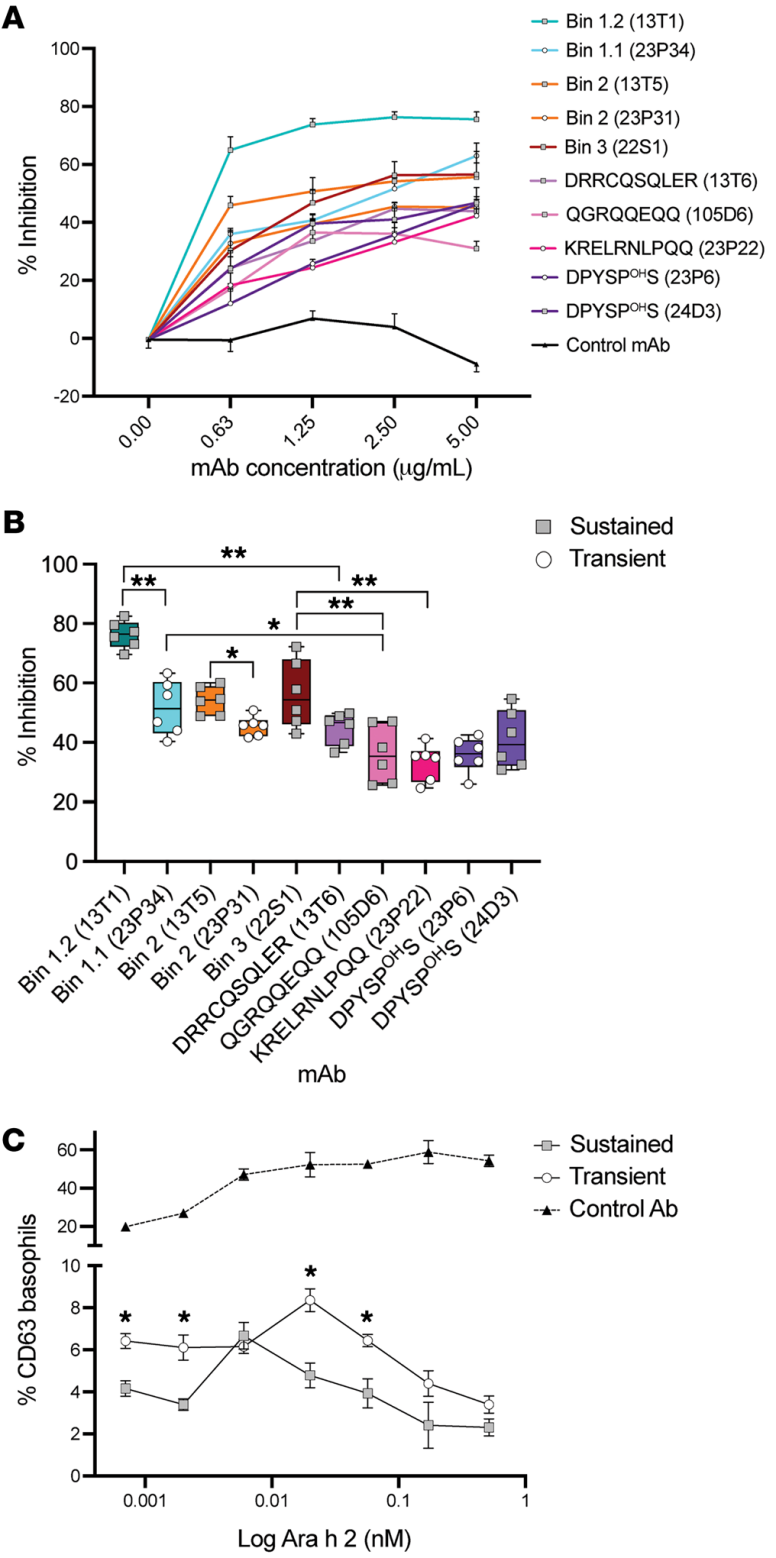
Allergen neutralization by Ara h 2 antibodies. (**A**) Antibody saturation curves with a competitive inhibition ELISA using Ara h 2 mAbs to evaluate inhibition of pooled serum IgE from 6 patients. Serially diluted Ara h 2 mAbs were added to the ELISA wells. Data are expressed as the mean ± SEM of 6 replicate wells. (**B**) Percentage of inhibition of pooled serum IgE by individual Ara h 2 mAbs normalized to serum IgE without antibody controls (shading by epitope). mAbs from transient responders (white circles) and sustained responders (gray squares) are represented. Data are expressed as the mean ± SEM of 6 replicate wells. Multiple comparisons were performed by ANOVA and corrected using the FDR; adjusted *P < 0.05 and adjusted **P < 0.001. (**C**) iBAT dose-response curve for Ara h 2 preincubated with a nonspecific control antibody (black triangles), sustained antibody mix (gray squares), or a transient antibody mix (white circles) measured by the percentage of CD63 upregulation on human basophils. Data are expressed as the mean ± SEM of 3 replicates. *P < 0.05, by Student’s *t* test.

To test our hypothesis that antibodies from sustained responders are uniquely able to block IgE binding to Ara h 2, we used peanut-allergy plasma pooled from placebo-treated OIT patients (*n* = 6) in an indirect competitive ELISA. We found that all epitope–specific mAbs blocked serum IgE binding, represented as a percentage of inhibition, in a dose-dependent manner compared with an Ara h 2–nonspecific control antibody (Figure 5A).

Antibodies recognizing epitopes 1.2 and 3, specific to sustained responses, significantly inhibited serum IgE binding to Ara h 2. Upon comparison of individual mAbs, we found that antibody 13T1 against the tolerant epitope 1.2 significantly inhibited more IgE binding than did antibody 23P34 against the epitope 1.1. Additionally, antibody 22S1, from epitope 3, also significantly inhibited IgE binding (Figure 5B).

On average, blockade of conformational epitopes decreased IgE binding significantly (*P* < 0.001), which was also significant when comparing colocalized conformational and sequential epitopes. In support of this observation, antibody 13T1 against epitope 1.2 significantly blocked more IgE binding than did antibody 13T6 against the sequential epitope ^30^DRRCQSQLER^39^. Antibody 23P34 significantly blocked more IgE binding than did antibody 105D6 against epitope ^121^QGRQQEQQ^128^. Antibody 22S1 significantly blocked more IgE binding than did antibody 105D6 against the ^121^QGRQQEQQ^128^ epitope or antibody 23P22 against the ^130^KRELRNLPQQ^139^ epitope (Figure 5B).

Altogether, antibody recognition of conformational epitopes, especially those epitopes specifically identified in sustained responders, significantly disrupted serum IgE interactions with Ara h 2, thereby neutralizing allergen effectively.

### Neutralizing Ara h 2–specific antibodies functionally suppress basophil activation.

To test the functional blocking capacity of these antibodies, we used the mAbs in an indirect basophil activation test (iBAT). We hypothesized that the recognition of unique and common epitopes by antibodies from sustained responders confers a greater ability to neutralize allergen, thereby suppressing IgE-mediated activation.

Basophils used in the iBAT were sensitized with peanut allergy plasma pooled from placebo-treated OIT patients (*n* = 6) and stimulated with natural Ara h 2 preincubated with a mixture of 4 antibodies designed to block the 4 discrete regions of Ara h 2 (Figure 1C and Supplemental Table 4). To compare the effect of epitope specificity of antibodies from sustained or transient responders, we used 13T1 (epitope 1.2) in the sustained mixture and compared it with 23P34 (epitope 1.1) in the transient mixture. For bin 2, we used 13T5 in the sustained mixture and compared it with 23P31 in the transient mixture. Since bin 3 was only present in sustained responders, we compared it with the sequential epitope ^130^KRELRNLPQQ^139^ present in the same region of Ara h 2. Finally, to block the DPYSP^OH^S epitope, we compared antibody 24D3 in the sustained mixture with 23P6 in the transient mixture.

The iBAT demonstrated suppression with allergen-specific antibody mixtures, with significantly greater (*P* < 0.05) suppression by antibodies from sustained responders compared with those from transient responders, illustrating the functional relevance of unique tolerance-associated epitope recognition in sustained responders to OIT (Figure 5C).

## Discussion

The mechanism behind antibody-mediated tolerance after treatment with immunotherapy for IgE-mediated food allergy has been unclear, despite the changes in the levels of serum Igs in patients receiving OIT. In this work, we defined the molecular determinants of neutralizing antibodies underlying clinical tolerance in peanut allergy. Our approach involved the use of recombinant Ara h 2–specific antibodies cloned from OIT-treated peanut-allergic patients categorized as either transient or sustained responders. Using these antibodies, we structurally characterized epitopes of Ara h 2 to elucidate the mechanism of antibody-mediated tolerance. We first defined and localized binding of 3 conformational and nonoverlapping epitope bins through a combination of epitope binning by BLI, chimeric proteins, and x-ray crystallography. Then, by comparing the binding of antibodies cloned from transient and sustained responders, we identified unique Ara h 2 epitopes associated with sustained, but not transient, responders. Antibodies recognizing these unique Ara h 2 epitopes more effectively disrupted allergen-IgE interactions and suppressed basophil degranulation. We found that the induction of unique Ara h 2–specific neutralizing antibodies during OIT was a mechanism that promoted the durability of allergic tolerance.

Previous work has focused on sequential, or linear, epitopes of Ara h 2 and other allergens using peptide microarrays (25–28); however, nonsequential, or conformational, epitopes have proven to be more challenging to characterize. Here, we describe what we believe to be 2 new intrahelical epitopes of Ara h 2 using x-ray crystallography. These nonoverlapping epitopes are located at opposite sides of the Ara h 2 structure, and each bind to multiple α-helices. Both epitopes also bind to heavy and light chain paratopes, highlighting the relevance of selective pressures on both heavy and light chains. As both of these conformational epitopes overlap with sequential epitopes, we were able to use antibodies recognizing these sequential epitopes as a tool to differentiate overlapping conformational epitopes within bin 1, leading to identification of epitope 1.2 as an epitope recognized solely by antibodies from sustained responders. Curiously, both epitope 1.2, recognized by antibodies 13T1 and 13A4, and epitope 3, recognized by antibodies 22S1, 06B1, and 27A4, were epitopes specific to antibodies from sustained responders.

Binding assays suggested that antibodies against conformational epitopes, including epitopes 1.1, 1.2, and 3, could also block antibodies specific to sequential epitopes. Previous work highlights the clinical relevance of sequential epitope recognition by IgE in peanut-allergic individuals as a diagnostic (29, 30) and prognostic tool (26, 28). The sequential epitopes of the mAbs studied in this manuscript have all been previously identified in peanut-allergic individuals (25–27, 31). In particular, the ^30^DRRCQSQLER^39^ epitope, within epitope 1.2, has been identified as a commonly recognized IgE epitope in several cohorts of both children and adults with peanut allergy (25, 26, 30). The ability of epitope 1.2–specific conformational antibodies to block widely occurring DRRCQSQLER-specific IgE antibodies probably contributes to its unique capacity for functional neutralization of allergen. In a similar fashion, the bin 3–tolerant antibodies also blocked both ^130^KRELRNLPQQ^139^ and ^121^QGRQQEQQ^128^ sequential epitopes (31), again demonstrating how unique targeting of conformational regions, capable of disrupting both conformational and sequential regions, are highly relevant to the neutralization capacity of these antibodies.

Our data suggest that allergen-specific antibodies recognizing these conformational epitopes on Ara h 2 promoted clinically sustained responses after OIT. Previous work consistently showed that OIT induces high levels of allergen-specific IgG and IgG4 antibodies in both desensitized groups and that these levels fall after avoidance (8, 32). As these levels diminish, the clinical relevance of neutralizing antibodies could increase in those with sustained responses, but further studies are needed. Moreover, the durability of clinical efficacy after OIT is probably a continuum, and in this study, categorization of responses as sustained or transient at discrete time points was a useful vehicle for comparing clinical efficacy. While our work is inherently limited, given the rarity of allergen-specific B cells and small samples of peripheral blood, the identification of multiple epitopes associated with tolerance suggests a possible explanation for the durability of clinical responses in IgE-mediated hypersensitivity. Our results may also support the concept that the decreased frequency or even absence of certain epitope-specific IgGs permits their epitope-specific IgE counterparts to continue to function in patients with transient responses to OIT. Additional questions about how these protective antibodies develop, the relationship between allergen-specific IgE and IgG, and modulation of these responses remain. Further studies are needed to better understand how these antibody-mediated mechanisms of tolerance relate to the previously identified changes in T cells, both effector and regulatory subsets (23, 33–38).

The insights here on how antibodies block certain epitopes on the immunodominant Ara h 2 have potential implications for the development of therapeutics for peanut allergy and other IgE-mediated food allergies. Peanut contains two 2S albumins, Ara h 2 and the highly homologous allergen Ara h 6 (39), to which many peanut-allergic individuals’ IgE also binds. Other common allergenic foods, such as tree nuts, sesame, and legumes, also contain allergenic 2S albumin proteins. This work does not directly show preservation of these epitopes after ingestion in peanut-allergic individuals, although previous work does suggest that intact Ara h 2 is systemically available after peanut ingestion (40–42). However, since mAbs studied in this effort were derived from the natural immune responses of patients undergoing OIT, our work suggests that these epitopes were preserved and presented to the immune system. Further studies to characterize the frequency of conformational Ara h 2 epitopes as well as their central role in inhibition of IgE-mediated allergic reaction are needed. Moreover, the protection offered by neutralizing antibodies targeting conformational epitopes may apply to other 2S albumins, which are major allergens, but may also apply to other food allergens induced by allergenic exposure either via immunotherapy or early introduction in sensitized children.

The possibility exists that the mechanism we described here extends beyond food allergens to other IgE-mediated allergies, such as aeroallergens. The induction of functionally relevant allergen-specific antibodies also occurs in immunotherapy for treatment of IgE-mediated allergic rhinitis (17). For example recently, neutralizing cat-specific antibodies that bind to conformational epitopes of Fel d 1 successfully treated allergic rhinitis in a proof-of-concept clinical trial, highlighting their therapeutic potential (43). Our work suggests that neutralizing antibodies capable of disrupting IgE-allergen interactions through specific epitope recognition may underlie the clinical durability of this intervention. Further studies on the clinical utility of neutralizing antibodies in peanut allergy are needed. Moreover, further studies on how the repertoires of allergen-specific IgE and IgG may evolve and relate, given the role of sequential class switching in the IgE repertoire (44–47), may also provide additional insights into clinical efficacy.

We also propose that neutralizing antibodies in food allergy may share structural similarities in antigen recognition to neutralizing antibodies against pathogens. The neutralizing antibody 13T1 has a notably long heavy and light chain CDR3 region, with 17 and 11 amino acids, respectively. In its crystal structure, the heavy chain Y103 residue, nestles into a positively charged pocket of Ara h 2 created by the intersection of multiple α-helices. This residue is also found in antibody 13A4, which binds to the same tolerant epitope and contains a distinct Y-containing motif of this public antibody. The longer CDR3 regions and the structural paratope adaptions of these tolerance-inducing neutralizing antibodies mirror those of neutralizing antibodies against pathogens such as HIV (48, 49). Interestingly, the chronic antigen exposure in OIT also mirrors the chronic antigenic exposure in HIV that is thought to be critical for the development of these unique neutralizing antibodies (50). These shared characteristics may contribute to the functional protective capacity of neutralizing antibodies.

By defining the molecular determinants of neutralizing antibodies against peanut allergens, this work will open new avenues for treatment of allergic diseases. Our work establishes attributes for potential therapeutic antibodies for the treatment of IgE-mediated food allergies. The structural characterization of Ara h 2 epitopes also informs the development of engineered hypoallergens for the treatment of IgE-mediated food allergies. More broadly, we believe this work introduces a central role for allergen recognition by neutralizing antibodies and shifts the therapeutic paradigm for allergic tolerance in IgE-mediated diseases.

## Methods

### Peanut OIT trial.

Peripheral blood was obtained from peanut-allergic participants enrolled in 2 clinical trials of peanut OIT. The first was a phase I/II open-label, randomized trial of peanut OIT (NCT01324401), with 30 peanut-allergic participants, aged 7–21 years, who had a clinical history of reaction to peanut within 1 hour of ingestion and either a positive skin-prick test (>8 mm wheal) or elevated peanut-specific IgE levels (CAP fluorescence enzyme immunoassay [FEIA] >10 kU/L; ImmunoCAP, Thermo Fisher Scientific). Participants were randomized for active treatment (peanut flour) or placebo (roasted oat flour) at a 3:1 ratio, with placebo-treated patients crossing into the active treatment arm after 6 months. The second clinical trial was a phase I/II, double-blind, placebo-controlled interventional study of peanut OIT (NCT01750879) with 40 peanut-allergic participants aged 12–55 years, who had a positive skin-prick test for peanut, a peanut-specific serum IgE level above 0.35 kU/L, a positive Ara h 2 IgE level above 0.35 kU/L, and a reaction to less than 443 mg peanut protein during the first double-blind, placebo-controlled food challenge. Participants were randomized to receive either active treatment (peanut flour) or placebo (roasted oat flour) at a 3:1 ratio.

In the active arm of both clinical trials, the participants underwent OIT, as previously described (13), and completed 3 months of a daily maintenance dose of 4 g, followed by an oral food challenge. After OIT, the participants avoided the allergen for either 1 or 3 months (first and second trials, respectively), followed by a second challenge. To characterize sustained or transient responses, we compared the change in the amount of peanut tolerated in the 2 challenges. In a sustained response, the amount of peanut tolerated remained the same compared with a transient response if the amount of peanut tolerated decreased.

### Specific Ig measurement.

Peanut- and antigen-specific Ig levels in plasma from peripheral blood of participants undergoing OIT was measured using a Phadia ImmunoCAP 1000 instrument (Phadia AB) according to the manufacturer’s instructions.

### Whole blood direct basophil activation.

Direct basophil activation was performed ex vivo, as previously described (13), using peripheral blood from participants undergoing peanut OIT. Briefly, using the Flow CAST assay, 50 μL fresh blood was stimulated with native Ara h 2 with a 5-fold dose response (Buhlmann Laboratories) and then stained with fluorescent anti-CCR3 and anti-CD3 antibodies, according to the manufacturer’s protocol for assessment by flow cytometry. Flow cytometric data were processed using a previously described pipeline with Bioconductor tools in R software (51). Activated basophils were identified as SSC^lo^CCR3^+^CD63^hi^. The CD63 cutoff was based on the medium control for each experiment. Data were analyzed using R, version 3.2.1, and GraphPad Prism, version 9.3.1 (GraphPad Software).

### Identification of Ara h 2–specific circulating B cells.

PBMCs were isolated by density-gradient centrifugation (Ficoll-Paque Plus, GE Healthcare) and cryopreserved in FBS with 10% DMSO. After the study outcomes were assessed, PBMCs (10 × 10^6^ to 25 × 10^6^ cells per sample) were thawed and washed with PBS. Ara h 2–specific B cells were selected by flow cytometry using a fluorescent natural Ara h 2–Alexa Fluor 488 multimer, as previously described (7), using CD3-APC (eBioscience, clone OKT3), CD14-APC (eBioscience, clone 61D3), CD16-APC (eBioscience, clone CB16), CD19-APC-Cy7 (BD Biosciences, clone SJ25C1), CD27-PE (BD Pharmingen, clone M-T271), CD38–Violet 421 (BD Biosciences, clone HIT2), and IgM-PE-Cy5 (BD Pharmingen, clone G20-127). Single Ara h 2–specific B cells, identified as Ara h 2^+^CD3^–^CD14^–^CD16^–^CD19^+^CD27^+^ B cells, were index sorted into individual wells in a 96-well plate by FACS (Aria II or Fortessa, BD Biosciences) and stored at –80°C for single-cell B cell receptor (BCR) amplification. Data were analyzed using FlowJo software, version 8.8.7 (Tree Star).

### Single-cell Ig gene amplification.

We conducted a previously described nested reverse transcription PCR (RT-PCR) protocol (7). Briefly, cells underwent heat lysis followed by amplification with random hexamers, before RT (SuperScript III Reverse Transcriptase, Invitrogen, Thermo Fisher Scientific). Using 2 nested PCRs, heavy and light chains were amplified using a modified primer set, as previously published (7). Gel electrophoresis was performed to detect PCR products. Successfully amplified products were Sanger sequenced (GENEWIZ) using the second nested PCR primers. Consensus sequences combining both the forward and reverse sequences were determined using pairwise alignment in Geneious, version 11.1 (Biomatters). These sequences were then analyzed using IMGT/V-BLAST (52, 53) to identify germline V, D, and J sequences with the highest identity and CDR3 amino acid sequences.

### Recombinant antibody cloning and production.

Seventy-four paired heavy and light chains were selected after sequencing as previously described for recombinant antibody cloning (7). Briefly, restriction sites were added to purified PCR products (QIAquick PCR purification, QIAGEN) before digestion with AgeI, BsiWI, Sall, and XhoI (New England BioLabs) for preparation for ligation with linearized vectors containing human IgG1, κ, or λ constant domains, respectively (gift from Michel Nussenzweig (The Rockefeller University, New York, New York, USA). Competent *E. coli* NEB5α bacteria (New England BioLabs) were transformed, followed by selection on Luria Broth (LB) (Miller) agar plates with 100 μg/mL ampicillin. Ampicillin-resistant clones were selected and screened for vector insertion with colony PCR using forward and reverse primers (19) followed by gel electrophoresis of PCR products. Sanger sequencing of plasmid DNA was validated by alignment to previous single-cell heavy and light chains (Geneious). Plasmid DNA (25 ng) from selected heavy and light chains were transfected into HEK293 T cells (ATCC, CRL-3216) using GenJet In Vitro DNA Transfection Reagent (SignaGen) or jetPRIME versatile DNA/siRNA transfection reagent (Polyplus). Supernatants were harvested from cells after culturing for 3 days with GenJet or for 2 days with jetPRIME at 37°C with 5% CO_2_ in serum-free HL-1 media (Lonza) supplemented with penicillin/streptomycin and 8 mM GlutaMAX (Gibco, Thermo Fisher Scientific). Ara h 2 specificity of recombinant antibodies was validated using commercial ImmunoCAP assays (Phadia) and/or BLI assays.

### Ara h 2 sequential peptide microarray.

Peptide microarrays were created using 15-mer linear synthetic peptides overlapping by 8 amino acids of the Ara h 2.0201 isoform immobilized on a microarray slide (PepStar, JPT Peptide Technologies). Full-length human IgG, human IgE, and mouse IgG were coimmobilized on the microarray slides as controls. After blocking (Superblock TBS T20, Pierce International), mAbs were diluted to 0.2 μg/mL and incubated on the slides for 1 hour at 30°C. A secondary Alexa Fluor 647 fluorescence-labeled anti–human IgG antibody (Jackson ImmunoResearch) at 1 μg/mL was then incubated for 1 hour at 30°C. Before each step and after secondary antibody incubation, the slides were washed with 50 mM TBS-buffer with 0.1% Tween-20, pH 7.2. The slides were scanned using a high-resolution laser scanner (Axon Genepix Scanner 4300, Molecular Devices) at 635 nm to obtain fluorescence intensity profiles for quantification by GenePix Pro 7 (Molecular Devices).

### BLI assays.

All BLI assays were performed on an Octet K2 or R2 Protein Analysis System (Sartorius). All assays were run at a plate temperature of 30°C and a shaking speed of 1,000 rpm unless otherwise noted. mAbs were diluted in kinetics buffer (Dulbecco’s PBS [DPBS] plus 1% [w/v] BSA plus 0.02% [v/v] Tween-20; Amresco) into a black flat-bottomed, 96-well plate (Greiner Bio-One). Sensors were regenerated at most 10 times with a 30-second cycle of regeneration in glycine 1.5 pH (Bio-Rad) and neutralization in kinetics buffer. All analysis was performed using Octet Analysis Studio Software, version 12.2 (Sartorius).

Purified mAbs were quantified using anti–human Fab-CH1 (FAB2G) biosensors (Sartorius). To create a standard curve for concentration calculation, a 3-fold serial dilution to 8 dilutions was repeated in triplicate using a commercially available IgG1 antibody (R&D Biosystems) in DPBS buffer (Corning). Response (nm) was measured after 1:1 curve fitting.

mAbs were measured by BLI after loading biotinylated native Ara h 2 ( 0.5 μg/mL in kinetics buffer, Indoor Biotechnologies) on streptavidin sensors (Sartorius) for 100 seconds. To determine the ideal binding concentration of mAbs, we performed binding curves on 12 purified, undiluted antibodies that were diluted 3-fold for an 8-series dilution. Each experiment included a reference sensor to normalize for any measurement drift and a reference well to account for any nonspecific binding. Affinities were measured using a minimum of 4 curves with a reference well, with a χ^2^ value of less than 3 and an *R^2^* value of greater than 0.95. Affinities were similar using BLI and surface plasmon resonance (SPR).

To identify additional antibodies that recognize sequential epitopes, biotinylated peptides were loaded on streptavidin sensors (Sartorius) for BLI. These biotinylated peptides were labeled with biotin and a hydrophilic linker (TTDS) on the N-terminus (BioTides, JPT Peptide Technologies). We loaded sensors with the peptides diluted to 0.5 μg/mL in kinetics buffer for 25 seconds. mAbs were then introduced for 150 seconds, and binding was measured by the response (nm).

Epitope binning of mAbs was performed using an in tandem assay on BLI to measure the response of secondary antibody binding after saturation of the primary antibody. Primary mAbs were diluted to 10 μg/mL and secondary mAbs to 5 μg/mL in kinetics buffer. Streptavidin sensors (Sartorius) were loaded with biotinylated natural Ara h 2 (Indoor Biotechnologies) at 0.5 μg/mL for 100 seconds. Primary antibodies were then loaded for 150–500 seconds, and after a 60-second baseline step, secondary antibodies were loaded for 150–300 seconds. Antibody binding was measured by the response (nm). Secondary antibodies were grouped into a separate epitope if binding was 0.16 nm or higher (≥4 times the SD of nonbinding antibodies). Analysis was performed using Octet Analysis Studio Software, version 12.2, and GraphPad Prism, version 9.3.1.

### mAb binding to chimeric 2S-albumins.

Recombinant 2S-albumins, Ara h 2.01, and Ara h 6 were produced using the expression of synthetic genes as previously described (24). The variant rAra h 2.Δ was obtained by replacing the sequence GRDPYSP^OH^SQDPYSP^OH^SP of Ara h 2.0201 by the dipeptide DS occurring in Ara h 6 and was previously validated in its ability to induce degranulation of peanut allergy serum–sensitized effector cell assays (24).

Competitive inhibition assays to measure binding of biotinylated 2S albumins to Ara h 2 mAbs were performed as previously described (24). Briefly, plates were coated with a mouse anti–human IgG1-Fc-CH2 mAb (Bio-Rad Laboratories, clone NL-16), followed by binding of 50 μL purified antibody (0.5–10 ng/mL) during an overnight incubation at 4°C. Subsequently, 25 μL of increasing concentrations of recombinant chimera ranging from 3.2 pM to 100 nM were added and incubated for 4 hours at room temperature, followed by addition of neutravidin labeled with AChE (2 EU/mL). Washes were performed between each step using 0.05% Tween-20 in 25 mM phosphate buffer at pH 7.4. The buffer (0.1% BSA, 0.15M NaCl, 0.01% sodium azide in 0.1 M phosphate buffer at pH 7.4) for dilutions of mAbs, biotinylated tracers, and inhibitors was also used to saturate the plate. Binding inhibition is represented as B/B0 to represent the amount of the labeled tracer in the presence of the inhibitor.

### Recombinant Ara h 2 production for crystallography.

Ara h 2.0101 (31–160) was fused with an N-terminal 6His-thioredoxin tag and a TEV cleavage site to create a gene construct called *HisTRX(TEV)A2* that was bacterially expressed and purified. *HisTRX(TEV)A2* was subcloned into pET32b with the NdeI and EcoRI restriction sites, followed by transformation into *E*. *coli* Origami B cells and plated onto LB agar plates containing antibiotics (100 μg/mL ampicillin, 50 μg/mL kanamycin, and 12.5 μg/mL tetracycline). A glycerol stock was prepared and inoculated into 25 mL Luria broth containing the antibiotics for overnight culture, which was transferred into 1 L Terrific broth with the same antibiotics. Cells were grown at 37°C until the OD_600_ reached 0.6 when 500 μM Isopropyl-β-d-thiogalactopyranoside (IPTG) was added to induce protein expression, and cells were incubated overnight at 18°C. Cells were harvested by centrifugation at 4,000*g* for 15 minutes, and the pellet was lysed by sonication in the resuspension buffer (500 mM NaCl in 25 mM Tris at pH 8.0). The soluble fraction was separated by centrifugation at 47,900*g* and loaded onto 5 mL Ni-NTA in a batch at 4°C. The resin was washed with the buffer 3 times, followed by batch elution with resuspension buffer containing 400 mM imidazole. Concentrated protein was loaded onto Superdex 200 26/60 equilibrated with resuspension buffer (500 mM NaCl in 25 mM Tris at pH 8.0) and the peak fractions, and kept at –80°C until use.

### Recombinant antibody expression for crystallography.

Heavy and light chain antibody plasmids (13T1 and 22S1) were prepared using the QIAGEN Plasmid Plus Giga Kit as described in the manufacturer’s manual. Recombinant antibodies were expressed using the ExpiCHO expression system (Thermo Fisher Scientific) with the Max titer protocol. ExpiCHO-S cells (500 mL, Thermo Fisher Scientific) were prepared in ExpiCHO expression media as a suspension culture using Thomson Optimum Growth flasks with 8% CO_2_ and 80% humidity at 37°C while shaking at 130 rpm at a density of 6 × 10^6^ cells/mL. Equal amounts (0.5 mg each) of heavy and light chain vectors were added into the final 20 mL OptiPRO SFM media and incubated for 5 minutes at room temperature. ExpiFectamine (1.6 mL) was added to 18.4 mL OptiPRO SFM media and incubated for 5 minutes at room temperature. These 2 media containing DNAs and ExpiFectamine were added to the ExpiCHO-STM cells (density of 6 × 10^6^ cells/mL) for incubation in a shaker for 22 hours (day 0). To enhance antibody expression, 3 mL ExpiCHO enhancer mixed with 80 mL ExpiCHO feed media was added to the culture. The cells were incubated with 5 % CO_2_ and 80% humidity at 32°C while shaking at 130 rpm (day 1). On the fifth day, an additional 80 mL ExpiCHO feed media was added to the culture. On the 14th day, cells were harvested by centrifugation at 500*g* for 10 minutes. The supernatant was then mixed with diatomaceous earth and filtered (0.2 μm).

### Recombinant antibody purification and Fab preparation.

The secreted antibodies were captured by Protein A resin (GoldBio) equilibrated with 0.1 M Tris pH 8.0, 500 mM NaCl, eluted with 0.1 M glycine pH 2.5, and 500 mM NaCl and neutralized with 1/10 volume of 1 M Tris pH 8.0. For the preparation of Fabs, the buffer of antibodies was exchanged using a Hitrap desalting column (Cytiva) equilibrated with the fresh digestion buffer (1× PBS with 0.02 M EDTA and 0.02 M l-cysteine, pH 7.0). Antibodies were digested with papain-immobilized resin (32 mg AB/mL settled resin; Thermo Fisher Scientific) in the digestion buffer overnight at 37°C. Fabs were collected from the flowthrough fractions after passing through the HiTrap rProtein A FF column equilibrated with 0.1 M Tris pH 7.5 and 0.5 M NaCl. Full-length antibodies and Fabs were analyzed using SDS-PAGE gel. The concentration of antibodies was estimated using an extinction coefficient of 1.4 (mg/mL).

### Crystallization and structure determination.

HisTRX(TEV)A2 protein was mixed with 22S1 Fab at a molar ratio of 1.2:1.0 and allowed to form a complex at room temperature for 30 minutes. The TRX tag in the complex was digested with TEV protease at 4°C, while dialyzing against the buffer 25 mM HEPES pH 7.4 and 150 mM NaCl for 18 hours. The complex was confirmed and purified with 16/60 Superdex 200 (GE Healthcare) equilibrated with the dialysis buffer. The 22S1Fab–Ara h 2 complex was mixed with an equal molar of 13T1 Fab and purified with Superdex 200 16/60. 22S1 Fab alone (10 mg/mL), 22S1 Fab–Ara h 2 complex, and 22S1 Fab–13T1–Fab–Ara h 2 complex were used for the crystal screening against the MCSG screens 1 through 4 (Midwest Center for Structure Genomics; Anatrace) at 4°C and at room temperature using sitting-drop vapor diffusion. Diffraction quality crystals of 22S1 alone were obtained from the condition containing 22S1Fab–Ara h 2 in sodium citrate tribasic dihydrate pH 5.6, 0 % v/v 2-propanol, and 20 % w/v PEG4000 at 4°C, however, no Ara h 2 was present in the crystal. Ternary complex crystals of 22S1Fab–13T1Fab–Ara h 2 were obtained from 0.05 M zinc acetate, 20% (w/v) PEG3350. The ternary complex crystals were further optimized in 0.1 M sodium acetate pH 5.5, 0.05 M zinc acetate, and 16% PEG 3350. We added 20%–25% ethylene glycol as a cryoprotectant for data collection. Data were collected at the Southeast Regional Collaborative Access Team (SER-CAT) 22-ID beamline at the Advanced Photon Source, Argonne National Laboratory (wavelength, 1.0 Å; temperature, 100 K) (54). For 22S1 Fab phasing, molecular replacement was performed with pertuzumab (PDB ID: 4LLY) as a search model (55) using the Phaser (56) module in Phenix (57). The 22S1–13T1–Ara h 2 complex structure was solved using 22S1 Fab as a model and Ara h 2 (Protein Data Bank [PDB] ID: 3OB4) (21). The model was refined using iterative cycles of refinement in Phenix and manual building in Coot (58). Ramachandran statistics are 97.3 % favored and 0.33 % outliers, respectively, as determined by Molprobity (59).

The 3D cartoons were made with PyMol (PyMOL Molecular Graphics System, version 2.0, Schrödinger). The surface electrostatic potential was calculated using APBS (60).

### Indirect competitive ELISA.

An indirect competitive ELISA was used to evaluate the ability of mAbs to disrupt peanut allergy serum IgE binding to Ara h 2. Microtiter plates were coated with 5 μg/mL natural Ara h 2 (Indoor Biotechnologies) overnight at 4°C and then blocked (PBST, consisting of 0.05% Tween-20 and 1% BSA) for 2 hours. Purified Ara h 2–specific mAbs (0.625–5 μg/mL) were added for 2 hours, followed by a 2-hour incubation with a 1:50 dilution of IgG-depleted peanut allergy pooled plasma from placebo-treated OIT participants (*n* = 6). For IgE detection, anti-IgE conjugated to HRP (1:10,000, Bethyl Laboratories) was added for 1 hour. Plates were washed with PBST between each step of the protocol, and all incubations were performed at room temperature, unless otherwise specified. For the colorimetric assay, 3,3′,5,5′-tetramethylbenzidine (TMB) substrate (BioLegend) was incubated for 45 minutes, and the reaction was stopped with 1 M phosphoric acid (Thermo Fisher Scientific). Absorbance was measured at 450 nm on a SpectraMax iD5 spectrophotometer (Molecular Devices) with SoftMax Pro 7.1 (Molecular Devices). Before analysis, the background was subtracted from OD values. The percentage of inhibition was calculated after normalization to the control (without a mAb).

### iBAT.

PBMCs from a healthy donor were isolated by density-gradient centrifugation (Ficoll-Paque Plus, GE Healthcare). Endogenous surface IgE was stripped using a previously published lactic acid stripping protocol (13, 61) (13.4 mM lactate, 140 mM NaCl, 5 mM KCl, pH 3.9), followed by neutralization with 12% Tris (pH 8). Pooled IgE from peanut allergy plasma isolated from placebo-treated participants (*n* = 6) was loaded onto basophils during a 1-hour incubation at 37°C to load IgE onto basophils. After incubation, the cells were washed with RPMI with 0.5% BSA and 2 μg/mL IL-3, and then stimulated with Ara h 2 (Indoor Biotechnologies) with mAb mixtures in basophil activation buffer (0.5% BSA, 2 mM CaCl_2_ and 2 mM MgCl_2_ in RPMI 1640 Media). Prior to stimulation, the Ara h 2 and antibody mixtures were preincubated at 37°C for 40 minutes in RPMI with 0.5% BSA, before addition to the cells. Each antibody mixture was composed of mAbs at a molar ratio of 0.6 mAb to Ara h 2 (Supplemental Table 4). Since there are multiple DPYSP^OH^S epitopes within the Ara h 2 molecule, which can activate basophils as a result of IgE cross-linking, a 3 times higher molar ratio of these antibodies was used in the mixture compared with the ratio of the other mAbs.

Experimental controls included stimulation with medium alone and anti-IgE stimulation (Bethyl Laboratories). After stimulation at 37°C for 30 minutes, ice-cold 0.2 M EDTA was added to stop degranulation. The cells were washed and resuspended in staining buffer (PBS plus 0.1% BSA) for staining with fluorescent antibodies using CD63-BV421 (BioLegend, clone H5C6), CD203c-PE (BioLegend, clone NP4D6), CD123-FITC (BioLegend, clone 6H6), and HLA-DR-APC (BioLegend, clone L243). Flow cytometry was performed on a CytoFLEX S (Beckman-Coulter). Degranulation was assessed as the frequency of CD63^hi^ basophils (singlet SSC^lo^CD123^+^HLA-DR^–^CD203^+^CD63^hi^). The CD63 cutoff was set on the medium-only stimulation control. Data were analyzed with FlowJo software, version 10.8 (TreeStar).

### Code availability.

The code used to analyze direct basophil activation testing is available at https://github.com/saritaupatil/AutoBAT/ (branch: Master, commit ID: d2ed5f63f347ef7d18a7a686c4dc95a841ece82f).

### Data availability.

Structural data are available through the PDB (ID: 8DB4).

### Statistics.

Continuous variables were summarized by the median and the SD. Comparisons between 2 groups were performed using either a nonparametric Mann-Whitney *U* test or an unpaired, 2-tailed Student’s *t* test. Comparisons between multiples groups were done using a Kruskal-Wallis ANOVA. For multiple comparisons among several nonparametrically distributed variables, *P* values were adjusted by controlling the FDR to not exceed 0.05 using the approach by Benjamini, Krieger, and Yekutieli. *P* values of less than 0.05 were considered significant. Box plots were plotted with the 25th and 75th percentiles for the lower and upper hinges, respectively. Statistical and graphical analyses were performed with GraphPad Prism, version 9.3.1. Amino acid sequence alignment was performed using R (4.1.1) package msa (62).

### Study approval.

Participants in both clinical trials were recruited with informed consent, and the studies were approved by the IRB of Mass General Brigham Healthcare (protocols 2010P000609 and 2012P002153).

## Author contributions

Co–first authors NAL and JM contributed significantly to this work, and the authorship order reflects the timeline of their project involvement. NAL isolated antibodies, led the production of recombinant antibodies, performed BLI assays and iBATs, conducted data analysis of antibody and clinical data, created the figures, and edited the manuscript. JM co-led the structural biology team, performed the structural studies, analyzed structural data, created figures, and edited the manuscript. TK performed ELISAs and iBATs, analyzed data, and edited the manuscript. CMR developed the high-yield antibody production methodology. KA, JZ, and OMR produced antibodies, performed assays, and edited the manuscript. AP led high-yield antibody production and antibody validation assays, interpreted data, and edited the manuscript. SH and BH conceptualized allergen chimeras, performed assays with the chimeric proteins, and edited the manuscript. WGS led the clinical trials (principal investigator of NCT01324401 and NCT01750879), collected samples, conducted direct BATs, conceptualized allergen-specific multimers, and edited the manuscript. JCL designed and directed antibody amplification studies and edited the manuscript. GAM and LCP conceptualized the structural biology approaches, led the structural biology team, contributed to data interpretation, and edited the manuscript. SUP conducted clinical trials (co-investigator), designed allergen-specific B cell multimers, isolated antibodies, performed antibody cloning and production, designed BLI assays, designed basophil activation, performed data analysis, and wrote and edited the manuscript.

## Supplementary Material

Supplemental data

## Version 1 01/17/2023

Electronic publication
