# Supplementary material for: Immunotherapy-induced neutralizing antibodies disrupt allergen binding and sustain allergen tolerance in peanut allergy
Source: J Clin Invest. 2023 Jan 17;133(2):e164501. doi: 10.1172/JCI164501 (PMC9843057; doi:10.1172/JCI164501)
Supplement: Supplemental data [file jci-133-164501-s169.pdf]

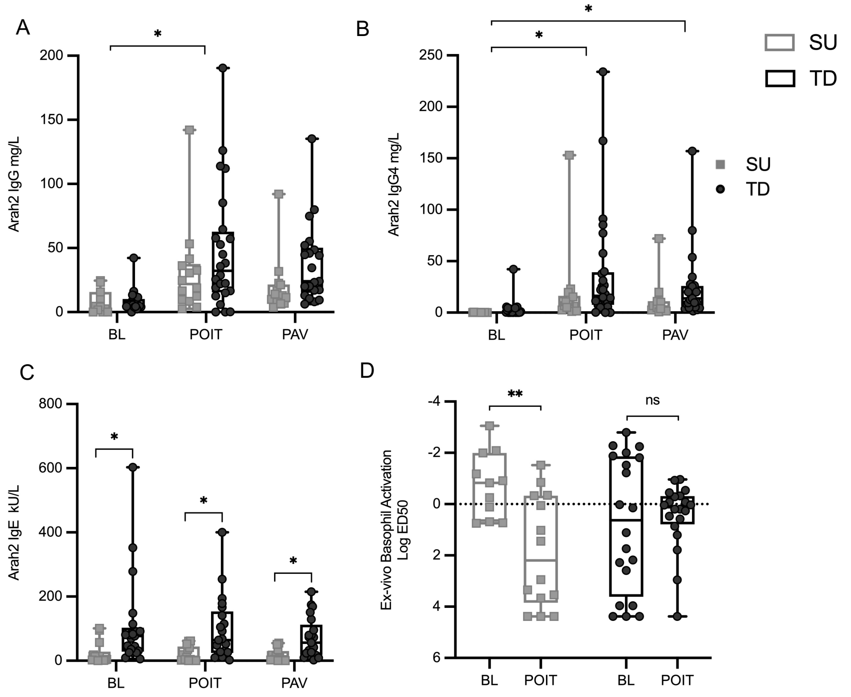

Supplementary Figure 1. Serum Ara h 2 antibody levels and ex vivo direct basophil sensitivity in peanut OIT.

Serum Ara h 2 specific IgG (A), IgG4 (B), and IgE (C) levels in sera from patients before OIT (BL), after OIT (POIT), and after 1-3 months of peanut avoidance (PAV) with both sustained and transient responses (adjusted *P<0.05, ANOVA test with FDR correction). (D) Sensitivity of ex vivo direct basophil activation to Ara h 2, measured as the log ED50 of the dose-response curve to Ara h 2 stimulation (**P<0.01, Mann-Whitney test). Error bars represent standard error.

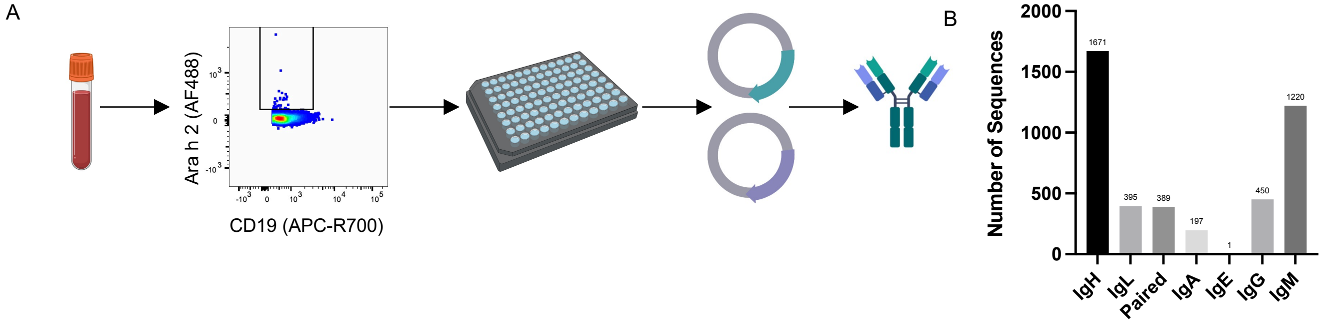

Supplementary Figure 2. Recombinant cloning of antibodies from Ara h 2 specific B cells. (A) Ara h 2 specific B cells were affinity selected from peripheral blood of OIT subjects by flow cytometry using an Ara h 2 multimer for single-cell sorting into a 96-well plate, where paired heavy and light chains were amplified, sequenced, and cloned into heavy and light chain plasmids for subsequent recombinant IgG1 antibodies. **(B)** Numbers of heavy, light, and paired sequences, along with isotyping of heavy chain sequences.

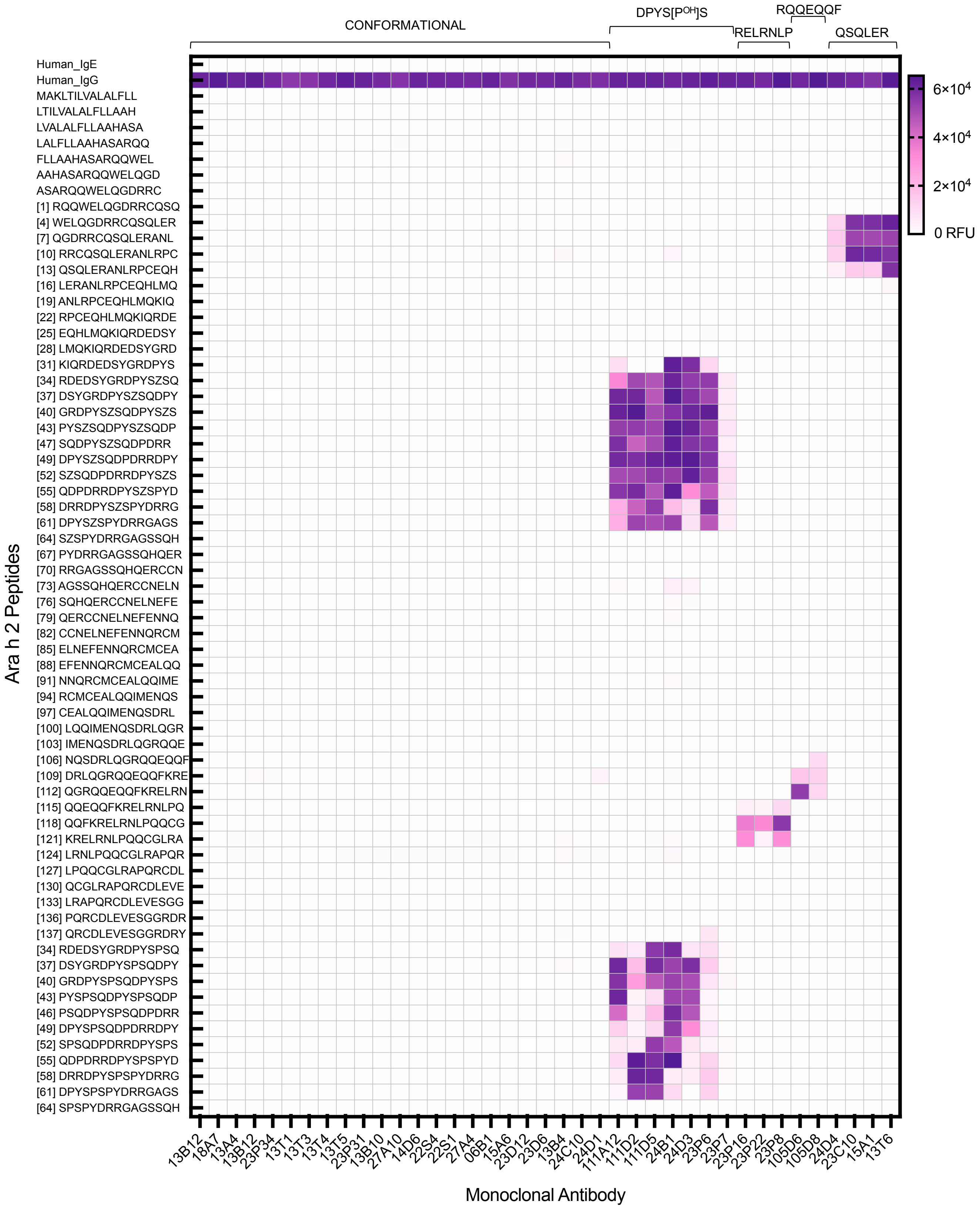

Supplementary Figure 3. Sequential epitope recognition by Ara h 2 specific monoclonal antibodies. Peptide microarray using overlapping peptides generated from Ara h 2.0201 isoform (left) and monoclonal Ara h 2 specific antibodies (bottom) showing binding (colorscale, relative fluorescence units). "Z" in the peptides denotes the hydroxylated proline (P^{OH}).

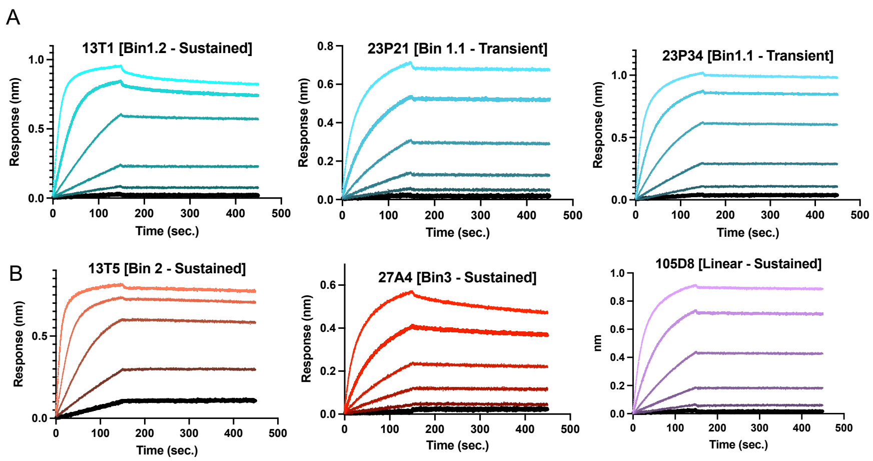

Supplementary Figure 4. Affinity of Ara h 2 specific monoclonal antibodies. (A) Biolayer interferometry binding curves for measurement of affinity. Representative monoclonal antibodies from sustained and transient responses and **(B)** Bin 1, 2, 3, and sequential recognizing monoclonal antibodies.

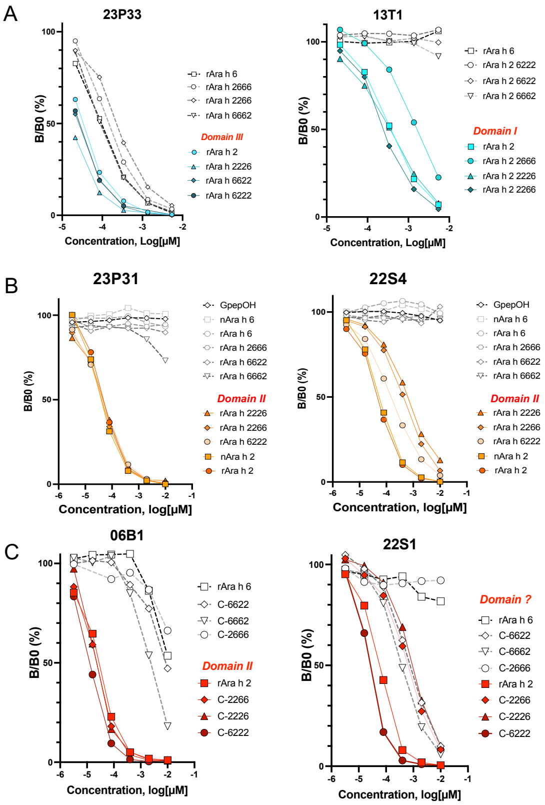

Supplementary Figure 5. Monoclonal antibody binding to chimeric proteins. Bin 1 **(A)**, 2 **(B)**, and 3 **(C)** specific monoclonal antibodies were evaluated using a competitive inhibition ELISA for binding to the chimeric proteins.

A

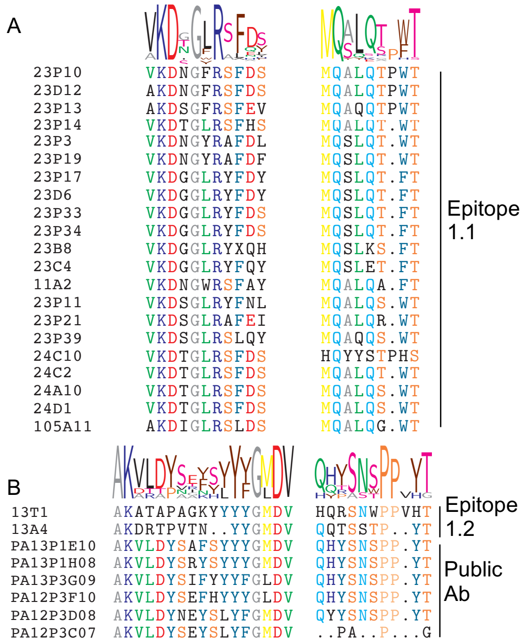

C

Identity	T1	13A4
	0.68	0.68
PA13P1E10	0.69	0.68
PA13P1H08	0.69	0.68
PA13P3G09	0.72	0.74
PA12P3F10	0.72	0.71
PA12P3D08	0.75	0.71
PA12P3C07	0.79	0.76

X Asp, Glu
X Arg, Lys, His
X Phe, Tyr, Trp
X Ala, Gly
X Cys, Met
X Ser, Thr
X Asn, Gln
X Leu, Val, Ile
X Pro

Supplementary Figure 6. Sequence alignment of Bin 1 antibodies. Multiple sequence alignment using ClustalW of IMGT-defined CDR3 regions of both heavy and light chains was performed for **(A)** epitopes 1.1 and **(B)** epitope 1.2 along with previously identified Ara h 2 IgE antibodies. **(C)** Identity of 13T1 and 13A4 to public antibodies.

Study	Subject	Age (years)	Sex	Race	Clinical Outcome	Peanut protein tolerated post-OIT (mg)	Avoidance (months)	Peanut protein tolerated post-avoidance (mg)
PNOIT1	965-06	7	Female	Asian	Sustained	5000	1	5000
PNOIT1	965-11	10	Male	>1	Transient	5000	1	3850
PNOIT1	965-13	10	Male	White	Sustained	5000	1	5000
PNOIT1	965-14	7	Male	White	Sustained	5000	1	5000
PNOIT1	965-15	8	Male	White	Sustained	5000	1	5000
PNOIT1	965-18	8	Female	White	Transient	5000	1	3850
PNOIT1	965-21	10	Male	White	Transient	5000	1	3850
PNOIT1	965-22	8	Male	White	Transient	3850	1	3850
PNOIT1	965-23	12	Male	>1	Transient	5000	1	3850
PNOIT1	965-27	7	Female	White	Sustained	5000	1	5000
PNOIT2	1037-01	16	Male	White	Sustained	4443	3	4440
PNOIT2	1037-22	19	Female	White	Transient	4443	3	1440
PNOIT2	1037-24	36	Male	White	Sustained	4443	3	4440
PNOIT2	1037-29	28	Male	White	Transient	4443	3	1440
PNOIT2	1037-33	16	Female	White	Transient	4443	3	1440

PNOIT2	1037-89	8	Female	White	Transient	4443	3	1440
PNOIT2	1037-93	11	Male	White	Transient	4443	3	1440
PNOIT2	1037-105	22	Female	White	Sustained	4443	3	4440
PNOIT2	1037-106	32	Female	White	Sustained	4443	3	4440
PNOIT2	1037-111	22	Female	White	Sustained	4443	3	4440

Supplementary Table 1. Clinical demographics and outcomes. Demographics and clinical outcomes are shown for the participants from both clinical trials of peanut OIT, from whom Ara h 2 antibodies were recombinantly cloned (PNOIT1 NCT01324401, PNOIT2 NCT01750879).

mAb	RRCQSQLER	QGRQQEQQ	KRELRNLPQQ	DPYSP^{OH}S
21U2	3.1139	0.021	0.0325	0.0073
22C8	0.0296	0.025	0.0269	1.8403
22D12	0.0238	0.0307	0.0334	3.2276
33A11	0.0168	0.0256	0.0298	2.1951
33B2	0.0217	0.0258	0.0305	1.7539
93B12	3.483	0.0179	0.0264	0.0204
93C9	0.0245	0.0276	0.0376	7.2609

Supplementary Table 2. Response rates of sequential antibodies to biotides on BLI. Monoclonal antibodies were bound to biotinylated Ara h 2 specific peptides loaded on streptavidin sensors in a BLI assay to identify antibodies binding to sequential epitopes.

	13T1Fab/Arah2/22S1Fab (PDB ID: 8DB4)
**Data collection**	
Space group	P1
Cell dimensions	
a, b, c (Å)	78.80, 92.18, 96.18
α, β, γ (°)	95.04, 106.031, 108.87
Resolution (Å)	50.0-2.3 (2.34-2.30) *
R_{sym} or R_{merge}	0.078 (0.772)
I / σI	20.8 (1.6)
Completeness (%)	91.4 (93.1)
Redundancy	3.8 (3.7)
**Refinement**	
Resolution (Å)	40.75-2.30 (2.34-2.30)
No. reflections	96,469
R_{work} / R_{free}	0.1899/0.2351
No. atoms	
Protein	1,991
Ethylene glycol/Zn^{2+}	4/9
Water	307
B-factors	

Protein	48.3
Ethylene glycol/Zn^{2+}	66.7/54.4
Water	51.0
R.m.s. deviations	
Bond lengths (Å)	0.004
Bond angles (°)	0.675

*Values in parentheses are for highest-resolution shell.

Supplementary Table 3. Data collection and refinement statistics. X-ray crystallography data collection parameters and refinement statistics for 13T1/Arah2/22S1.

mAb	*Patient*	*Outcome*	*Epitope*	*Affinity*
13T1	13	Sustained	1.2	1.8×10^{-10}
13T5	13	Sustained	2	$<1.0 \times 10^{-12}$
22S1	22	Sustained	3	9.2×10^{-11}
24D3	24	Sustained	DPYSP^{OH}S	4×10^{-10}
23P34	23	Transient	1.1	7.2×10^{-11}
23P31	23	Transient	2	1.9×10^{-12}
23P22	23	Transient	KRELRNLPQQ	2.4×10^{-11}
23P6	23	Transient	DPYSP^{OH}S	2.1×10^{-9}

Supplementary Table 4. Composition of antibody mixtures. Antibody characteristics and clinical outcomes of the monoclonal Ara h 2 specific antibodies used in indirect basophil activation for comparison of sustained and transient antibody mixture.
